# A limited set of molecular biomarkers may provide superior diagnostic outcomes to procalcitonin in sepsis

**DOI:** 10.1186/cc11727

**Published:** 2012-11-14

**Authors:** RB Brandon, M Thomas, RA Brandon, D Venter, J Presneill, J Lipman, J Morgan, B Venkatesh, J Sackier, A Sutherland

**Affiliations:** 1 Immunexpress Pty Ltd, Brisbane, Australia; 2 Immunexpress Inc., Seattle, WA, USA; 3 Mater Health Services, Brisbane, Australia; 4 The University of Queensland and Royal Brisbane & Women's Hospital, Brisbane, Australia; 5 Princess Alexandra Hospital, Brisbane, Australia

## Background

Differentiating the systemic inflammatory response syndrome (SIRS) from sepsis is very important to clinicians. Procalcitonin (PCT) has been studied extensively as a marker of sepsis; however, its clinical utility remains uncertain [1]. Alternative approaches involving analysis of circulating biomarkers using gene expression (GE) show promise [2]. The primary objective of this study was to compare the diagnostic performance of a GE biomarker set, SeptiCyte^® ^Triage, with PCT in a mixed patient population.

## Methods

The dataset was derived from two clinical trials conducted across four tertiary care settings in Australia between 2008 and 2011 (ACTRN12610000465055). Critical care patients (*n *= 87) were enrolled if they fulfilled the 1992 Consensus Statement [3] for sepsis, severe sepsis or septic shock. Postsurgical patients were included as an infection-negative systemic inflammatory cohort (*n *= 31), with 71 healthy controls (HC) for comparison. Blood samples were collected within 24 hours of the surgical procedure or upon admission to ICU for sepsis patients. PCT was measured using a commercially available assay kit (Brahms PCT) and GE assessed using Affymetrix GeneChip Human Exon 1.0 ST arrays, where a set of biomarkers were identified *a priori*. A Support Vector Machine algorithm was used to calibrate the molecular biomarkers with respect to specific clinical groups. Bootstrap and permutation tests were performed on the difference in area under the receiver operating characteristic curves (AUC ROC) for PCT and crossvalidated posterior probabilities.

## Results

**Figure 1:**
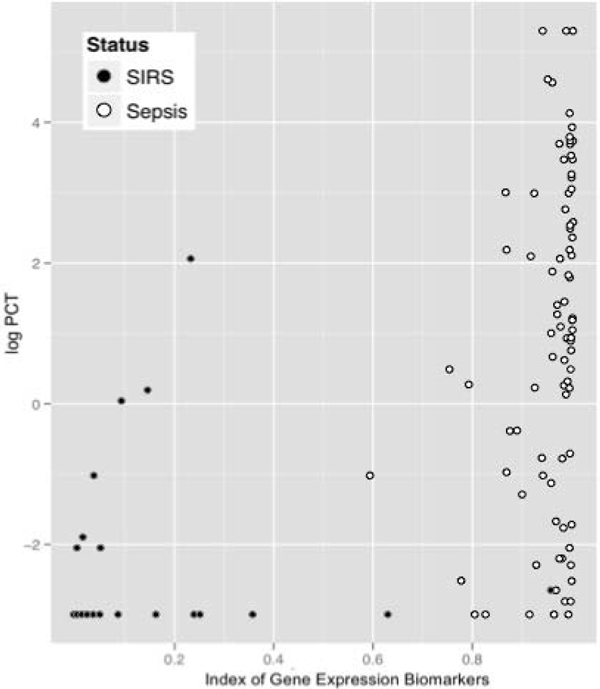
**Plot of log PCT (*y *axis) and index of SeptiCyte^® ^Triage gene expression biomarkers (*x *axis) for 31 SIRS (black) and 87 sepsis (white) patients demonstrating the ability of each technology to differentiate these conditions**.

The SeptiCyte^® ^Triage molecular biomarker set was significantly better than PCT at differentiating non-infectious systemic inflammation from all sepsis groups with an AUC ROC of 99.1% versus 90.8% (95% CI of difference = 4%, 16%), respectively. The differentiating ability of PCT and the SeptiCyte^® ^Triage biomarker set is demonstrated in Figure 1. The 99.1% AUC ROC of the biomarker set could be maintained when using a set of less than 10 genes, important for transferring the biomarkers to a rapid assay format. As a quality step, a classifier was developed that differentiated HC from sepsis with an AUC ROC of 100%.

## Conclusion

In comparison with the diagnostic performance of PCT, a limited set (<10) of molecular biomarkers potentially has a significantly better sensitivity and specificity profile for the detection of sepsis within a mixed systemic inflammatory patient group.
